# Rattusin structure reveals a novel defensin scaffold formed by intermolecular disulfide exchanges

**DOI:** 10.1038/srep45282

**Published:** 2017-03-27

**Authors:** Hye Jung Min, Hyosuk Yun, Sehyeon Ji, Ganesan Rajasekaran, Jae Il Kim, Jeong-Sun Kim, Song Yub Shin, Chul Won Lee

**Affiliations:** 1 Department of Chemistry, Chonnam National University, Gwangju 61186, South Korea; 2 Department of Pharmaceutical Cosmetics, Kwangju Women’s University, Gwangju 62396, South Korea; 3 Department of Medical Science, Graduate School and Department of Cellular and Molecular Medicine, School of Medicine, Chosun University, Gwangju 61452, South Korea; 4 Department of Life Science, Gwangju Institute of Science and Technology, Gwangju 61005, South Korea

## Abstract

Defensin peptides are essential for innate immunity in humans and other living systems, as they provide protection against infectious pathogens and regulate the immune response. Here, we report the solution structure of rattusin (RTSN), an α-defensin-related peptide, which revealed a novel C_2_-symmetric disulfide-linked dimeric structure. RTSN was synthesized by solid-phase peptide synthesis (SPPS) and refolded by air oxidation *in vitro*. Dimerization of the refolded RTSN (*r*-RTSN) resulted from five intermolecular disulfide (SS) bond exchanges formed by ten cysteines within two protomer chains. The SS bond pairings of *r*-RTSN were determined by mass analysis of peptide fragments cleaved by trypsin digestion. In addition to mass analysis, nuclear magnetic resonance (NMR) experiments for a C15S mutant and *r*-RTSN confirmed that the intermolecular SS bond structure of *r*-RTSN showed an I-V’, II-IV’, III-III’, IV-II’, V-I’ arrangement. The overall structure of *r*-RTSN exhibited a cylindrical array, similar to that of β-sandwich folds, with a highly basic surface. Furthermore, fluorescence spectroscopy results suggest that *r-*RTSN exerts bactericidal activity by damaging membrane integrity. Collectively, these results provide a novel structural scaffold for designing highly potent peptide-based antibiotics suitable for use under various physiological conditions.

Defensins are cysteine-rich antimicrobial peptides (AMPs) that play crucial roles in the innate immune system by providing protection against infectious pathogens and regulating the immune response^1^,^2^,^3^,^4^. Defensins are widely expressed in various living organisms, such as mammals, plants, fish, birds, insects, and fungi. Mammalian defensins have been classified into three subgroups: α-, β-, and θ-defensins, based on their amino acid sequences and patterns of disulfide (SS) bond connectivity^5^ (Fig. 1). All defensin structures contain a β-sheet fold that is stabilized by three SS bonds formed by six cysteine residues^6^. α-defensins are composed of 29–35 amino acids with three intramolecular SS bonds in an I-VI, II-IV, III-V arrangement^7^,^8^. Human α-defensins (HDs) 1–4, also known as human neutrophil peptides (HNPs), were first discovered in neutrophils^9^ and were also found to be expressed in natural killer (NK) cells^10^, monocytes, and some T lymphocytes^11^. HDs 5 and 6, expressed in Paneth cells of the small intestine, play an important role in protecting the intestinal stem cells within the crypt^12^,^13^. β-defensins (comprised of 38–42 amino acids) contain three intramolecular SS bonds in an I-V, II-IV, III-VI arrangement^7^,^14^. Although genome-based analyses have identified 28 human and 43 mouse β-defensin genes^15^, only a few have been isolated and studied at the protein level to date. Human β-defensins (HβDs) 1–4 are predominantly secreted by epithelial cells and the male reproductive tract^3^,^16^,^17^. HβDs 5–6 appear to be specifically expressed in the epididymis^18^. θ-defensin isolated from the leukocytes of the rhesus macaque is the only known mammalian N to C cyclized defensin, and it consists of 18 amino acids with the six-cysteine motif that forms three intramolecular SS bonds in an I-VI, II-V, III-IV arrangement^19^,^20^. Although mRNA transcripts of θ-defensin are present in human bone marrow, spleen, thymus, testis, and skeletal muscle, a premature stop codon aborts their translation^21^.

Mammalian defensins exhibit broad-spectrum antimicrobial activity against a wide variety of bacteria, fungi, viruses, parasites, and also antibiotic-resistant bacteria. For example, α-defensins exhibit antibacterial activity against Gram-positive and Gram-negative bacteria, but with different levels of efficiency^22^. HβDs exhibit bactericidal activity *in vitro* against several bacteria^23^ and also inhibit various fungal species, including *Candida*^24^. Some studies have reported that α-defensins are also able to inhibit human immunodeficiency virus (HIV) and herpes simplex virus (HSV) infections *in vitro*^25^. Despite intensive studies on the antimicrobial activity of defensins, the mechanistic aspects of their activities have remained unclear. There are several reports that defensins disrupt microbial membranes by forming carpets and pores and by inducing membrane instability^1^. Moreover, defensins also interact with the cell wall precursor lipid II and inhibit cell wall synthesis^26^. In addition to this functional diversity, the activity and efficiency of defensins against pathogens are modulated by several factors, such as incubation time, dose, pH, and salt concentration. In particular, some AMPs including defensins are salt-sensitive. For instance, β-defensins show different levels of sensitivity to physiological salt concentrations^27^.

In addition to α-, β-, and θ-defensins, β-hairpin AMPs adopt a β-sheet structural fold formed by two antiparallel β-strands stabilized by multiple intramolecular SS bonds (Fig. 1). AMPs are cationic and amphipathic peptides with membrane-binding properties and a broad range of activity against bacterial and fungal pathogens. Owing to their broad antimicrobial activity and resistance to physiological factors, β-hairpin AMPs have become a useful structural scaffold for the development of peptide-based antibiotics^28^,^29^.

The α-defensin-related peptide rattusin (RTSN), previously identified by genome-wide computational analysis of the rat genome^30^, has been recently studied at the protein level^31^. RTSN is preferentially expressed in Paneth cells of the distal small intestine in rats. The putatively matured RTSN, consisting of 31 amino acid residues (Fig. 1), exhibits highly potent antimicrobial activity against Gram-negative and Gram-positive bacteria, including antibiotic-resistant bacteria. Moreover, RTSN exhibits low cytotoxicity to mammalian cells, but retains its antimicrobial activity in the presence of physiological salt concentrations, suggesting that RTSN can be a valuable antimicrobial agent^31^. Furthermore, although RTSN shares a highly conserved signal sequence and prosequence with mammalian α-defensins, RTSN consists of five unusual cysteines with a distinctive spacing pattern instead of the six-cysteine motif typically found in the canonical mammalian defensins, indicating that RTSN can adopt a unique structural scaffold that has not been observed previously. In this study, we synthesized the functionally active refolded RTSN (*r*-RTSN) peptide and investigated its structural details, including SS bond pairings and the three-dimensional (3D) structure. The results suggested that the *r*-RTSN structure exists as a novel homodimeric scaffold formed by five intermolecular SS bonds. Moreover, we examined the mechanism of *r*-RTSN bactericidal activity using fluorescence experiments. The mechanistic studies indicated that *r*-RTSN might exhibit antimicrobial activity by disrupting microbial membranes. Collectively, these results indicate that *r*-RTSN can provide a novel structural scaffold that can be used to design highly potent peptide-based antibiotics.

## Results

### Preparation of *r*-RTSN

The linear precursor of RTSN, synthesized by solid-phase peptide synthesis (SPPS), was refolded in refolding buffer. Figure 2a shows the reverse phase-high performance liquid chromatography (RP-HPLC) analyses of RTSN before and after the refolding process. The observed mass of linear RTSN consisting of 31 amino acid residues was a deconvoluted *m/z* value of =3649.7 (calculated *m/z* = 3650.3) (Suppl. Fig. 1(a)). After oxidative refolding of linear RTSN, we obtained a product with a deconvoluted *m/z* value of 7288.4 that corresponded to the mass of the dimeric form of RTSN consisting of ten oxidized cysteines (Fig. 2b) (Suppl. Fig. 1(b)). These results indicated that *r*-RTSN contained five SS bonds in a homodimeric form.

### Determination of SS bond pairings of *r*-RTSN

**Figure 1:**
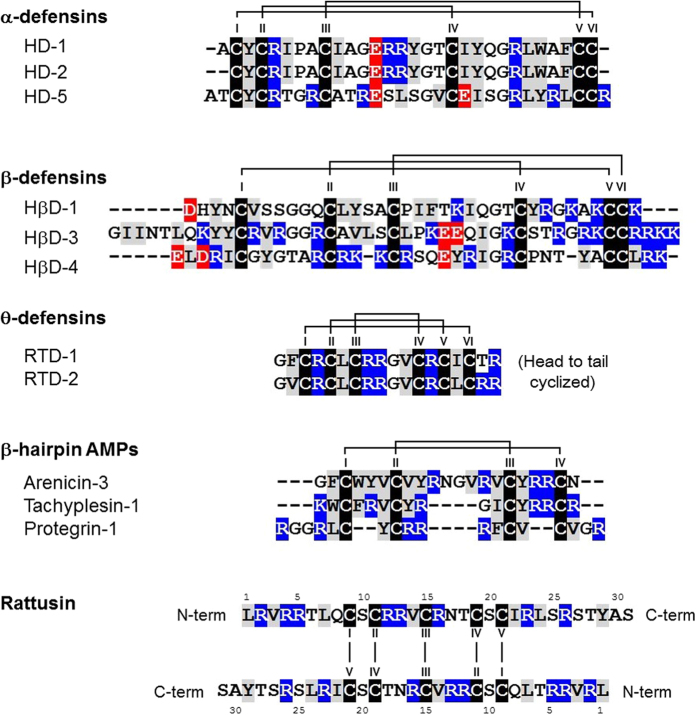
Amino acid sequences of α-, β-, and θ-defensins and β-hairpin antimicrobial peptides with their disulfide (SS) bond connectivity with solid lines. Cysteine, hydrophobic, positively charged, and negatively charged residues are colored with a shade of black, gray, blue, and red shadow, respectively. The amino acid sequence of dimeric rattusin is shown. Cysteine order is indicated in Roman numerals.

To determine the SS bond pairings of *r-*RTSN, we performed proteolytic digestion in combination with mass spectrometry analysis^32^. Since RTSN is an arginine-rich peptide (8 arginine residues in the monomer) (Fig. 1), we digested *r*-RTSN using trypsin, which can cleave the carboxyl side of arginine and lysine. The fragments resulting from trypsin digestion were separated and analyzed by RP-HPLC and liquid chromatography-mass spectrometry (LC-MS) (Fig. 3a). Four peptide fragments from the trypsin-digested *r*-RTSN were separated and their masses were determined by LC-MS analyses (Suppl. Fig. 2). Based on the comparison of the fragment masses with those of the RTSN primary sequence, we have proposed the peptide sequences of the fragments and their possible SS bond pairings (Fig. 3b) as follows: Fragment 1 (F1) is a dimeric short peptide (V^14^-C^15^-R^16^) connected by an intermolecular SS bond between the Cys^III^-Cys^III’^. Fragment 2 (F2) corresponds to a short peptide sequence of L^24^-S^25^-R^26^. Fragment 3 (F3) was identified as the C-terminal fragment consisting of S^27^-T^28^-Y^29^-A^30^-S^31^. Fragment 4 (F4) was the largest peptide with a mass of 1602.8. The possible structures of F4 (F4-1 and F4-2), proposed by the fragment masses and SS bond pairings, are shown in Fig. 3b. The results suggested that cysteine residues Cys^I^ or ^II^ with Cys^IV^ or ^V^ form intramolecular (F4-1) or intermolecular (F4-2) SS bonds. However, we were unable to distinguish between Cys^I^ and Cys^II^ and between Cys^IV^ and Cys^V^ using the enzyme fragmentation and mass analysis, since there are no cleavage sites between Cys^I^ and Cys^II^ and between Cys^IV^ and Cys^V^. Subsequently, it was confirmed that Cys^III^ and Cys^III’^ are clearly involved in dimer formation by an intermolecular SS bond (F1 of Fig. 3b). However, it was not clear whether the other cysteine residues (I, II, IV, and V) contributed to intramolecular or intermolecular SS bond formation in the *r*-RTSN structure. Therefore, we prepared a Cys^III^-substituted RTSN mutant (C15S), which could not form the Cys^III^-Cys^III’^ intermolecular SS bond. We hypothesized that if only Cys^III^ participates in the intermolecular SS bond formation (F4-1 structure of Fig. 3b), the refolded C15S RTSN will have a 3D structure similar to that of each protomer of *r*-RTSN, since the other cysteines (I, II, IV, and V) form only intramolecular SS bonds and do not affect monomeric RTSN folding and structure (Suppl. Fig. 3a). As a result, the refolded C15S mutant will exhibit an NMR spectrum similar to that of *r*-RTSN because *r*-RTSN is folded into a symmetric homodimer, which produces a monomer-like NMR spectrum (Fig. 4). On the other hand, if the other cysteines (I, II, IV, and V) also participate in additional intermolecular SS bonds (F4-2 structure of Fig. 3b) along with Cys^III^-Cys^III’^, the C15S mutant cannot be folded into the native structure (Suppl. Fig. 3b), which produces a different NMR spectrum for C15S compared to that of *r*-RTSN. The C15S mutant was refolded in the same refolding buffer used for *r*-RTSN. The refolded C15S mutant displayed a decrease in mass (by 4 Da) compared to the mass of the C15S mutant (Suppl. Fig. 4), indicating that all four cysteines in the C15S mutant were completely oxidized. We acquired the 2D total correlation spectroscopy (TOCSY) NMR spectrum^33^ of the refolded C15S mutant. (Suppl. Fig. 5). Interestingly, the well-dispersed cross peaks of *r*-RTSN were shifted within a narrow range for the C15S mutant in the TOCSY spectrum. In particular, the NMR cross peaks from all cysteines were clustered near 8.3 ppm for the backbone amide protons and near 4.5 ppm for the alpha protons (Suppl. Table S1) (cross peaks depicted as squares in Suppl. Fig. 5). These chemical shift ranges were close to those of the random coil structure^34^, indicating that the C15S mutant was not properly folded and that it exhibited disordered structural characteristics. These data suggest that the F4-2 structure (Fig. 3b) may be the actual structure for the F4 fragment. To confirm this, we determined the 3D structure of *r*-RTSN using solution NMR spectroscopy.

### NMR structure calculation of *r*-RTSN

The solution structure of *r*-RTSN was determined using constraints derived from 2D nuclear Overhauser effect (NOE) spectroscopy (NOESY) spectrum^35^,^36^ experiments and SS bond constraints. Complete proton resonance assignments for *r*-RTSN were determined using the traditional 2D NMR sequential assignment procedure. The 2D NOESY spectrum of *r*-RTSN shows the number of cross peaks corresponding to those of monomeric RTSN (only 30 residues, except for the first residue) (Fig. 4), indicating that *r*-RTSN forms a symmetric homodimer structure. The amino acid spin system was identified based on scalar coupling patterns observed in the DQF-COSY^37^ and TOCSY spectra. The identified spin systems were ordered along the primary sequence of RTSN through sequential NOEs in the 2D NOESY spectrum. Figure 4 shows the NH-C^α^H fingerprint region of the NOESY spectrum, which contains sequential *d*_αN_(*i, i* + *1*) connectivity. The initial structure of the *r*-RTSN was calculated using Cyana 2.1^38^ with automatic NOE assignment with only III-III’ SS bond restraints, and then the Cyana structures were further refined using Xplor-NIH^39^ with intermolecular SS bond restraints of the F4-2 structure. The β-hairpin region of *r*-RTSN was confirmed using hydrogen/deuterium exchange experiment and NOE pattern between β-strands (Suppl. Fig. 6). The NMR restraints and structural statistics for *r*-RTSN are summarized in Table 1. The sets of SS bond constraints (I-IV’, II-V’, III-III’, IV-I’, and V-II’) (Suppl. Fig. 7a) or (I-V’, II-IV’, III-III’, IV-II’, and V-I’) (Suppl. Fig. 7b) were possible in the F4-2 structure since we were unable to distinguish between Cys^I^ and Cys^II^ and between Cys^IV^ and Cys^V^ using enzyme fragmentation and mass analysis, then these constraints were included in the Xplor-NIH refinements. With the SS bond constraints shown in Suppl. Fig. 7a, Xplor-NIH refinement was terminated at an early stage before generating PDB template, probably since sulfur atoms in cysteine residues (Cys9, Cys19, Cys9′, and Cys19′) are too close each other to form proper disulfide bonds (Suppl. Fig. 8). These atom and/or bond clashes may produce too high energy in the Xplor-NIH refinement, and then the program was terminated. On the other hand, with the set of SS bond constraints of Suppl. Fig. 7b, the refined structures were well-converged and gave low root mean square (RMS) deviations and violations (Table 1). In addition, the Cyana calculation with the set of SS bond connectivity (I-V’, II-IV’, III-III’, IV-II’, and V-I’) (Suppl. Fig. 7b) exhibited more assigned NOE peaks and long-range distance restraints, but showed low values in both the target function and the RMSD in the final structure (Suppl. Table S2). Although the Cyana structure with the set of SS bond connectivity (I-IV’, II-V’, III-III’, IV-I’, V-II’) (Suppl. Fig. 7a) showed lower RMSD value than the other (Suppl. Table S2), the lower RSMD was probably due to too close atom contacts (Suppl. Fig. 8), which resulted in a higher target function than the other. Therefore, the Xplor-NIH refinement with the SS bond connectivity was terminated at an early stage. Taken together, Cyana results also imply that the SS bond connectivity of Suppl. Fig. 7b is the preferred SS bond structure for the *r*-RTSN.

### Structural description of *r*-RTSN

Figure 5 shows the structure of *r*-RTSN determined by solution NMR spectroscopy. The *r*-RTSN is a homodimeric structure with two identical protomers (Fig. 5a). Each protomer in the dimeric structure is composed of two β-strands, consisting of residues 9–12 (β1) and 18–21 (β2) (Fig. 5b). The protomer forms a β-hairpin structure with a seven-residue hairpin loop and flexible tails at both the N- and C-termini. Four cysteines (I, II, IV, and V) are located within the β-strands and Cys^III^ is placed in the middle of the hairpin loop. The dimerization of *r*-RTSN is formed by the four intermolecular SS bonds (I-V’, II-IV’, IV-II’, and V-I’) at the interface of the β-sheet in each protomer in an antiparallel fashion (Fig. 5b). An additional intermolecular SS bond (III-III’) connects the hairpin loop (Fig. 5b). The *r*-RTSN adopts a C_2_-symmetric covalently linked dimer, which is connected by five SS bonds, and the overall topology of *r*-RTSN exhibits a cylindrical array similar to that of β-sandwich folds (Fig. 5c). The core of the inter-protomer interface is made by the side chains from eight cysteines, forming four intermolecular SS bonds (Fig. 6a,b). The SS bond of III-III’ seems to stabilize the hairpin loop at the top of the molecule (Fig. 6c), which may also contribute to the overall structural stability, since the C15S mutant could not be folded into the native structure. RTSN is a highly basic peptide having a net charge of +8 (Fig. 1). All positively charged residues are arginines that are distributed on the surface throughout the molecule (including the N- and C-terminal tails and the hairpin loop region), which makes the surface highly positive (Fig. 7a). In addition, rattusin also contains seven hydrophobic residues in the monomeric sequence (Fig. 1). Some of these hydrophobic residues form a small hydrophobic patch on the surface. This surface characteristic is also found in several AMPs, such as θ-defensins and β-hairpin peptides (Fig. 1), as well as in α-helical AMPs. A Dali^40^ search for tertiary structure similarity did not identify any protein or peptide exhibiting structural similarity with *r*-RTSN. Furthermore, this unique structural fold of *r*-RTSN was not found in any other defensin peptide.

### Antimicrobial mechanism of *r*-RTSN

To confirm the antimicrobial efficiency of *r*-RTSN, we determined the minimal inhibitory concentration (MIC) value against the Gram-negative *Escherichia coli (E. coli*). The determined MIC was 8 μM, which is slightly greater than previously reported data (~4 μM against *E. coli* O157:H7)^31^. This small difference may result from differences in the bacterial species and/or experimental conditions. We further examined the mechanism of antimicrobial activity of *r*-RTSN by dye leakage and membrane depolarization. LL-37 and melittin, membrane-targeting (pore-forming or disrupting) AMPs, were used as positive control peptides. Buforin-2, an intracellular-targeting (non-membrane-targeting) AMP, was used as a negative control peptide. First, to determine if bacterial membranes can be preferentially targeted by *r*-RTSN, membrane perturbation was examined by measuring calcein release from negatively charged EYPE/EYPG(7:3) large unilamellar vesicles (LUVs). The *r*-RTSN induced dye leakage from EYPE/EYPG(7:3) LUVs in a dose- and time-dependent manner (Fig. 8a). LL-37 (3 μM) and *r*-RTSN (16 μM) induced 90% and 80% dye leakage from EYPE/EYPG(7:3) LUVs within 1000 seconds (16.6 min), respectively. Melittin induced 100% leakage within a few seconds at a concentration of 1 μM. Next, the ability of *r*-RTSN to permeabilize the membrnes of intact *Staphylococcus aureus* cells was determined using the membrane potential-sensitive dye DiSC3(5). Under the influence of a membrane potential, the dye DiSC3(5) can concentrate in the cytoplasmic membrane, resulting in a self-quenching of fluorescence. Upon disruption of the membrane potential, the dye dissociates into the buffer, causing an increase in fluorescence intensity. As shown in Fig. 8b, the fluorescence intensity of DiSC3(5) was strongly quenched due to the dye that accumulated in the membrane. After the signal was stable for 400 seconds, *r*-RTSN was added (arrow in Fig. 8b). The *r*-RTSN depolarized the bacterial cytoplasmic membrane in a dose- and time-dependent manner (Fig. 8b). Similar to LL-37, the addition of *r*-RTSN caused a gradual increase in fluorescence intensity due to a collapse of the ion gradients that generate the membrane potential. Melittin induced complete depolarization of the cytoplasmic membrane of *S. aureus* within a few seconds at a concentration of 1 μM. However, buforin-2 did not induce any dye leakage or membrane depolarization.

## Discussion

In this study, we report the solution structure of *r*-RTSN, which revealed a novel C_2_-symmetric dimeric structural scaffold. RTSN has been known as an α-defensin-related peptide, since it shares a highly conserved signal sequence and presequence with mammalian α-defensins. However, in contrast to α-defensins, the unique cysteine composition and dimeric formation observed in our study suggested that RTSN forms a distinct structural fold, which has not been observed yet in other known defensins.

**Figure 2:**
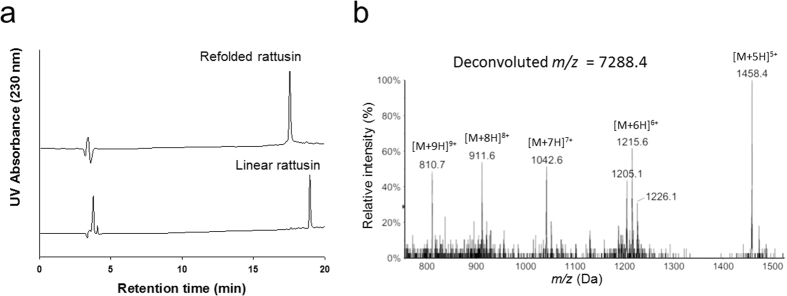
RP-HPLC and LC-MS analysis of the rattusin (RTSN) peptide. (**a**) The linear precursor and refolded RTSN (*r-*RTSN) were analyzed by RP-HPLC. (**b**) ESI-LC-MS ionization pattern of the *r-*RTSN.

We synthesized and refolded the active form of the RTSN peptide, which showed an antimicrobial activity consistent with previously reported data. As shown in Fig. 2a, the linear precursor of RTSN was oxidized into a major product in the refolding buffer. The *r*-RTSN was eluted earlier than the linear RTSN in the RP-HPLC column. This indicated that the refolded form was apparently more hydrophilic than the linear precursor, which may imply that polar and/or charged residues protrude from the surface of *r*-RTSN. As shown in the surface structure (Fig. 7a), *r*-RTSN developed a predominantly basic surface due to the presence of arginine residues. This agrees with the earlier retention time of *r-*RTSN compared to that of the linear RTSN.

**Figure 3:**
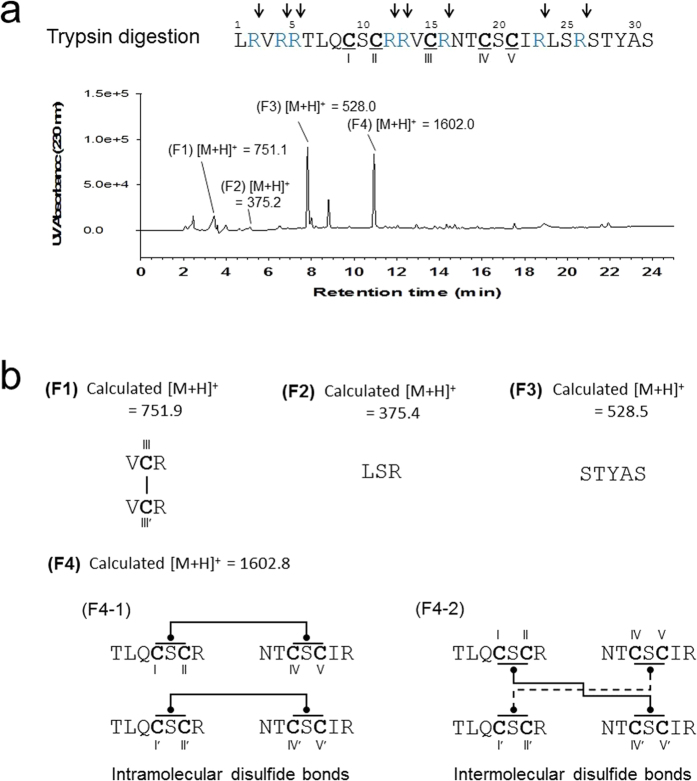
(**a**) Trypsin digestion and LC-MS analysis of refolded rattusin (*r-*RTSN). Trypsin cleavage sites are indicated by arrows on the sequence of RTSN. The digested peptide fragments were separated by RP-HPLC and their masses were analyzed by LC-MS and indicated on each peak (F1~4). (**b**) Peptide sequence and SS bond structure of the fragment proposed by mass analysis and primary sequence of RTSN. The proposed SS bond structure of F4 fraction is either F4-1 or F4-2.

**Figure 4:**
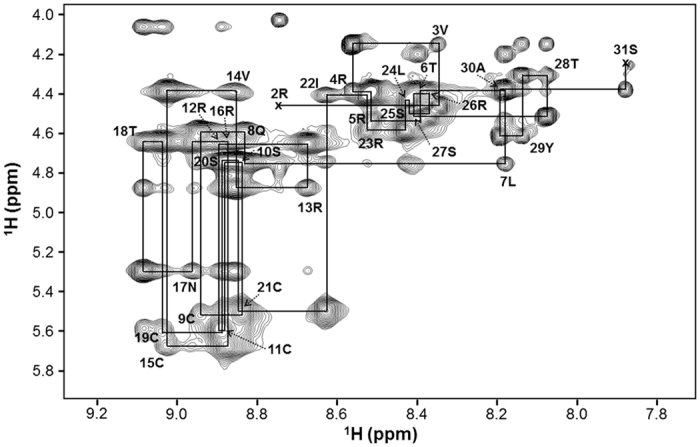
Sequential *dα*N(*i, i* + *1*) nuclear Overhauser effect (NOE) connectivities for refolded rattusin (*r-*RTSN) in a ^1^H 2D NOESY spectrum observed with a mixing time of 200 ms at 298 K. Intra-residue NH-C^α^H cross peaks are labeled with the residue number of RTSN using standard single-letter amino acid abbreviations. The first residue of RTSN was not observed in the NOESY spectrum.

**Figure 5:**
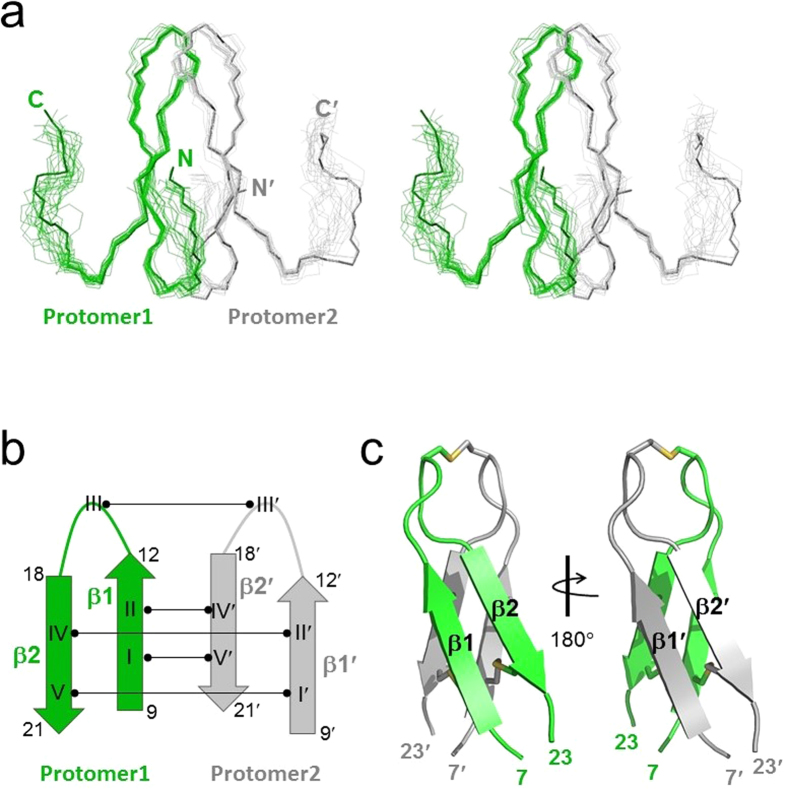
NMR solution structure of refolded rattusin (*r-*RTSN). (**a**) Stereo-pair of backbone heavy atoms (N, C^α^, and C’) for the 20 converged line structures of *r-*RTSN. N(N’) and C(C’) indicate N- and C-terminal positions, respectively. (**b**) Schematic diagram of secondary structure and SS bond connectivities of *r-*RTSN. Residue numbers of β-strands are indicated and cysteines numbers are labeled in Roman numerals. (**c**) Lowest energy ribbon structure of *r-*RTSN. The β-strands are labeled and SS bonds are shown as yellow sticks. Only the structural region (from 7 to 23 residues) of *r-*RTSN is shown.

**Figure 6:**
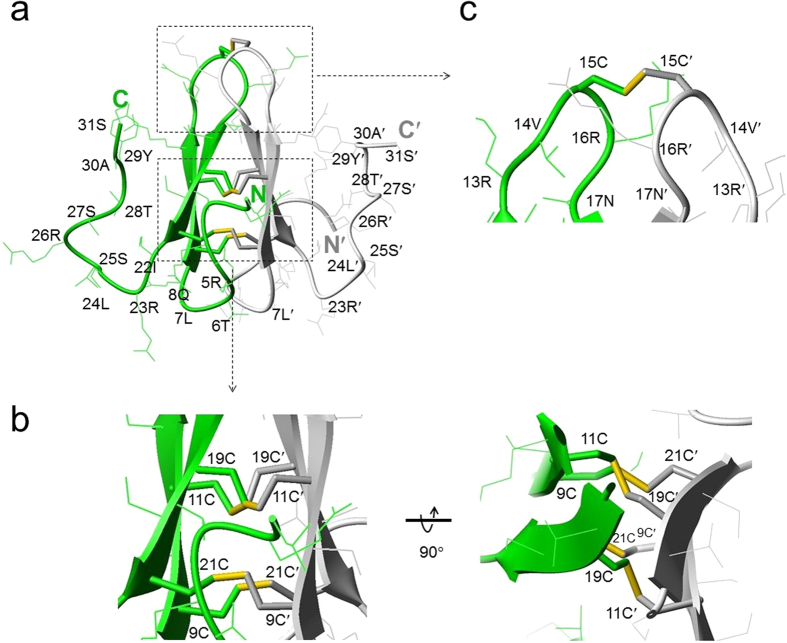
(**a**) Ribbon diagram with side chains of refolded rattusin (*r-*RTSN). All side chains are labeled with their residue number and single-letter amino acid abbreviations. SS bonds are shown as yellow sticks. (**b**) Intermolecular interface and core structure formed by four intermolecular SS bonds (9C-21C’, 11C-19C’, 19C-11C’, and 21C-9C’). (**c**) Intermolecular SS bond of 15C-15C’ at the hairpin loop of *r-*RTSN.

The primary sequence of RTSN contains an unusually odd number of cysteine residues, which strongly implies that *r*-RTSN will retain at least one intermolecular SS bond in the native form (note that two cysteines form a single SS bond, and then the complete SS bond formation requires an even number of cysteines). The observed mass of *r*-RTSN indicated that RTSN refolded into a dimeric form containing five SS bonds using ten cysteines from the two monomeric linear precursors. In a previous study, Patil *et al*. also found that synthetic RTSN refolded into a dimeric form that showed efficient antibacterial activity^31^ similar to the characteristics of *r*-RTSN observed in this study. They also proposed that RTSN forms a homodimer and/or oligomer through intra- and/or intermolecular SS bonds, and that the SS bond pairings may be random. However, we obtained a predominantly oxidized product in the refolding buffer and *r*-RTSN was homogeneous as a single component, as confirmed by RP-HPLC and NMR experiments. After trypsin digestion, only a few fragments exhibiting specific masses were detected by LC-MS (Fig. 3a), which also supported that a single component was produced after oxidative refolding, but not random SS bond formation.

The unique cysteine spacing pattern and dimeric formation made it difficult to determine SS bond pairings in *r*-RTSN. To determine the SS bond pairings of *r*-RTSN, we utilized enzyme digestion and mass analysis. We clearly identified the intermolecular SS bond linked by the Cys^III^-Cys^III’^ in the F1 fragment (Fig. 3b). However, the SS bond arrangement in the F4 fraction could not be identified in this experiment. In general, NMR experiments are able to provide a variety of structural information for proteins and peptides. Thus, in addition to mass analysis, NMR experiments of C15S mutant and the 3D structure of *r*-RTSN revealed the complete SS bond pairings within the *r*-RTSN.

Covalently linked dimer formation in AMPs has been reported. The cryptdin-related sequence (CRS) peptides, a family of AMPs identified from mouse intestinal tissue, also show conserved signal and prosequences with rodent α-defensins^41^. They consist of 9 or 11 cysteines in their mature primary sequences, thus forming homo- and heterodimers using intermolecular SS bonds *in vivo* and *in vitro*. It has been suggested that these varied dimerizations of CRS peptides allows an expansion of the repertoire that increases the diversity of the innate defense system. Recently, Wommack *et al*. found that human defensin 5 (HD5), an α-defensin expressed in human small intestinal Paneth cells, forms a disulfide-linked covalent dimer, which adopts a C_2_-symmetric β-barrel-like structure, as determined by solution NMR spectroscopy^42^. Dimerization occurred through two intermolecular SS bonds between cysteines 5 and 20′, and 20 and 5′ (II-IV’ and IV-II’), which are located at the hydrophobic interface (Suppl. Fig. 9a), suggesting that this unique structural fold not only presents a new example of the structural diversity for cysteine-rich AMPs, but also shows their increased proteolytic stability and antimicrobial potency. In addition to the CRS and HD5, the structure of *r*-RTSN also provides a new example of the disulfide-linked dimeric defensin structure, although the functional and structural importance of *r*-RTSN dimerization should be determined.

Bactericidal mechanisms of AMPs involve either disrupting microbial membranes or targeting intracellular components. Most antimicrobial peptides act on membranes by mainly causing membrane lysis by barrel stave, toroidal pore, or carpet-like mechanisms^43^,^44^,^45^. No single mechanism can be defined for all peptides^46^. In general, the oligomerization of peptides on the microbial membrane surface may be a crucial step in their antimicrobial mechanism. For instance, noncovalent dimerization or oligomerization of α- and β-defensins has been described and it seems to be associated with the modulation of their antibacterial activity. Rajabi *et al*. suggested that HD5 self-associates into a noncovalent dimer using a hydrophobic interaction between the second β-strands^47^. This hydrophobic dimerization interface was crucial for its function. It has also been reported that the noncovalent dimerization of β-defensin 3 enhanced its antimicrobial activity^14^. Previous studies demonstrated that the main target of AMPs was the cell membrane. In our case, *r-*RTSN induced a considerable dye (calcein) leakage from bacterial membrane-mimicking PE/PG liposomes in a dose- and time-dependent manner (Fig. 8a). *r-*RTSN can cause the membrane depolarization of intact *S. aureus* cells in a dose- and time-dependent manner (Fig. 8b). The dissipation of membrane potential caused by *r-*RTSN for *S. aureus* cells is comparable to that caused by the lytic peptide gramicidin D, a short very hydrophobic β-sheet peptide that can form transmembrane pores and ionic channels (Fig. 8b)^48^. The dissipation of membrane potential may be involved in channel or pore formation which allowed the passage of ions or larger molecules, thus leading to cytoplasmic content leakage and cell death^49^. Furthermore, the gradual increase in dye release and dissipation of membrane potential suggested that *r-*RTSN exerts bactericidal activity by damaging membrane integrity such as acyl chain perturbation, membrane defects, and/or membrane thinning rather than membrane disruption^50^.

**Figure 7:**
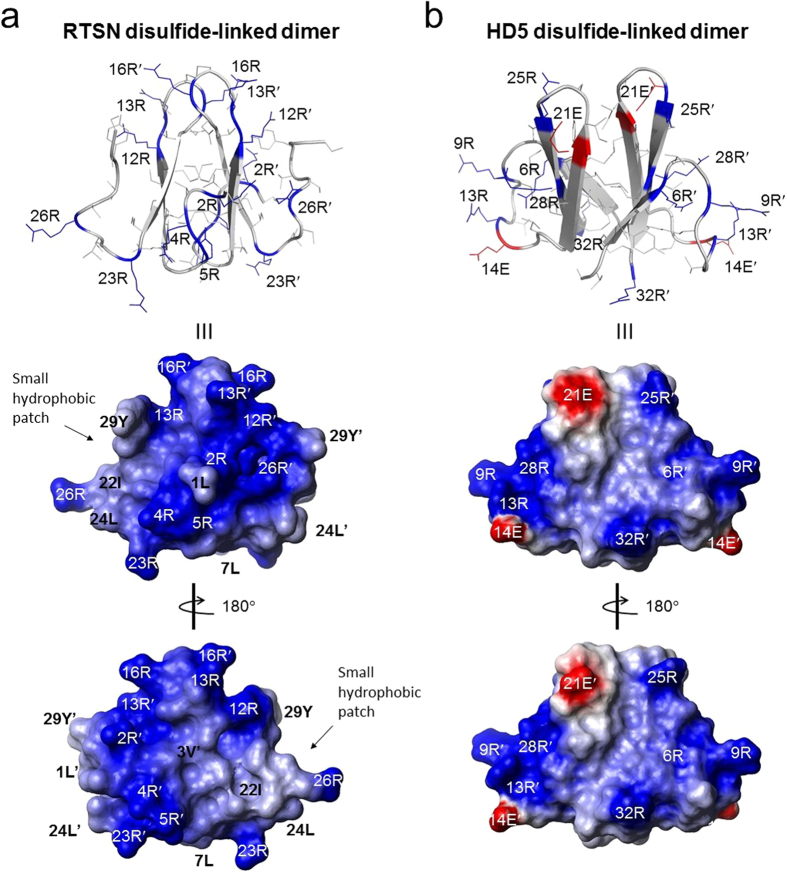
Surface structural characteristics of refolded rattusin (*r-*RTSN) (**a**) and dimeric HD5 (**b**). Positively and negatively charged residues are indicated in blue and red, respectively. The charged and hydrophobic residues are labeled on the side chain and surface structures. A small hydrophobic patch on the surface structure of *r*-RTSN is indicated.

**Figure 8:**
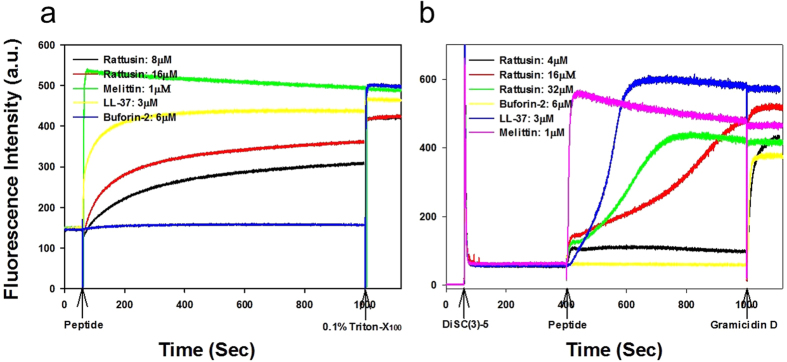
(**a**) Dose- and time-dependent peptide-induced calcein release from calcein-entrapped negatively charged EYPE/EYPG (7:3, w/w) LUVs. (**b**) Dose- and time-dependent membrane depolarization of *Staphylococcus aureus* by peptides.

The amphipathic structure of AMPs is a general structural feature consisting of a positively charged region interacting with the polar head group of lipids. The hydrophobic region of peptides inserts into the hydrophobic interior formed by membrane lipid tails, which then changes the membrane structure. RTSN effectively exhibits antibacterial activity against both Gram-negative and Gram-positive bacteria even in the presence of NaCl and Mg^2+^ 31. The highly basic *r*-RTSN may efficiently interact with negatively charged phospholipid membranes. In addition, covalently linked dimerization probably facilitates peptide oligomerization on the bacterial surface, even in the presence of salts, since the dimeric form of RTSN was found to be more active than the reduced monomeric RTSN in the presence of NaCl^31^. These data suggested that the dimeric form of RTSN could diminish the inhibitory effects of salts as a part of their antibacterial activity. Based on the surface properties of *r*-RTSN, the basic surface characteristics likely contribute to its salt-insensitive antibacterial activity. This may result from the fact that the highly positive surface is forced to effectively interact with the bacterial membrane surface even at high salt concentrations. In contrast to *r*-RTSN, the antibacterial activity of HD5 was completely inhibited by 100 mM NaCl^31^. HD5 has a net charge of +4 (6 arginines and 2 glutamates) and exhibits a combination of negative and positive charges on the surface (Fig. 7b). As an additional example, protegrin-1, a β-hairpin peptide from porcine leukocytes, is a highly cationic peptide (6 arginines) (Fig. 1) that displays a potent and broad-spectrum antimicrobial activity even at high salt concentrations (300 mM NaCl)^51^. These structural variations of AMPs seem to be responsible for their functional diversity. Interestingly, the structural topology of the protomer of *r*-RTSN resembles that of protegrin-1, exhibiting positive charge distribution with arginine residues and a β-hairpin fold (Suppl. Fig. 9d). However, the hairpin loop of protegrin-1 is much shorter and more compact than that of RTSN (4 vs. 7 residues), and the SS bond structures are very different (intramolecular vs. intermolecular). Further studies on the structural and functional determinants of *r*-RTSN and its related peptides will provide a structural basis for the development of peptide antibiotics.

In conclusion, here, we presented the structure of *r*-RTSN, which reveals a novel structural scaffold in cysteine-rich AMPs. The unique structure of *r*-RTSN provides a new addition to the examples of structural diversity in the defensin family. Therefore, the dimerization of defensins, including RTSN, may also provide the structural diversity of the peptides and increase in activity and stability of the AMPs in an *in vivo* environment. The disulfide-linked dimeric scaffold and surface property of *r*-RTSN can be a structural basis for the design of a highly potent AMP in physiological conditions. Moreover, further investigations of the structural and functional determinants of *r*-RTSN will provide valuable information for the development of peptide-based antibiotics.

## Methods

### Peptide synthesis

The linear precursor of RTSN was synthesized by standard solid-phase Fmoc chemistry, starting from Fmoc-Ser(tBu)-Wang resin, using a variety of blocking groups for amino acid protection. Linear RTSN was cleaved from the resin with a mixture of TFA (trifluoroacetic acid), H_2_O, ethanedithiol, phenol, and thioanisole (percentage volume ratios 82.5/5/2.5/5/5). After stirring for 3 h at 25 °C, the solution was cooled and mixed with a 10-fold volume of diethyl ether precooled to 0 °C. The resultant precipitates were washed thoroughly with diethyl ether. The linear RTSN was purified using a preparative RP-HPLC system (LC-6AD, Shimadzu) with C_18_ column, 20 × 250 mm, and purity and mass were confirmed by analytical RP-HPLC (Shimadzu LC-10ADvp) and a triple-quadrupole equipped electrospray ionization (ESI)-LC-MS (API2000, AB SCIEX). The mobile phase components were 0.05% TFA in water and 0.05% TFA in acetonitrile.

### Oxidative refolding

The linear RTSN were diluted to a final peptide concentration of 2.5 × 10^−5^ M and subjected to oxidative SS bond formation at 4 °C in 20 mM Tris-HCl (pH 8.0), 50 mM NaCl. When close to equilibrium, the reactions were stopped by lowering the pH to approximately 3.0 with TFA. The refolding procedure was monitored by analytical RP-HPLC and LC-MS.

### Trypsin digestion and mass analysis

The SS bond pairings of the *r-*RTSN were determined using trypsin digestion and LC-MS analysis. The *r-*RTSN was digested with trypsin in 50 mM Tris-HCl (pH 8.0) for 16 h at 25 °C. Trypsin-digested peptide fragments were separated on a C_18_ column equipped with the Shimadzu LC-10ADvp system and their molecular masses were analyzed using LC-MS.

### NMR spectroscopy

NMR measurements were carried out on a Bruker AVANCE 600 spectrometer equipped with an XYZ gradient triple-resonance probe. The samples used for proton 2D NMR experiments were 1 mM samples dissolved in 50 mM sodium phosphate buffer (pH 6.5) containing 10% D_2_O at 298 K. TOCSY spectra were recorded using a MLEV-17 pulse scheme with isotropic mixing times of 60 and 90 ms. NOESY spectra were obtained with mixing times of 100 and 200 ms. Suppression of the solvent resonance in both the NOESY and TOCSY spectra was achieved using the WATERGATE scheme^52^. DQF-COSY spectra were collected with water pre-saturation. NMR data processing and analyses were performed using NMRPipe^53^ and NMRView^54^.

### Structure calculation

NOE resonance assignments and initial NOE constraints were obtained from CYANA 2.1 with CANDID^55^. All NOE constraints were manually confirmed during CYANA calculations. Hydrogen bonds for the β-strand regions of *r-*RTSN were added to facilitate the automated assignment of additional NOE restraints by CANDID. For each SS bond, we used three distance constraints, S(*i*)-S(*j*), S(*i*)-C^β^(*j*), and S(*j*)-C^β^(*i*), whose target values were set to 2.02 ± 0.02, 2.99 ± 0.5, and 2.99 ± 0.5 Å, respectively^56^. The initial 100 structures were refined by a simulated annealing protocol with Xplor-NIH. Iterative refinement and editing of the distance constraints based on the NOESY spectra to remove incorrect and ambiguous assignments reduced the number of constraints. The final 20 structures with the lowest energy were chosen for analysis and were deposited in the Protein Data Bank (accession code 5GWG). All images were prepared with MOLMOL^57^ and Pymol^58^ programs.

### Determination of MIC

The antibacterial activity of *r-*RTSN was tested in sterile 96-well 200 μL plates as follows. Aliquots (100 μL) of *E. coli* cell suspension at 4 × 10^6 ^CFU/mL in 1% peptone were added to 100 μL of the sample solutions (serial 2-fold dilutions in 1% peptone). After incubation for 16 h at 37 °C, the MIC was determined by visual examination on the basis of the lowest concentration of sample solution in cells with no bacterial growth. The Gram-negative *E. coli* (KCTC 1682) was procured from the KCTC at the Korea Research Institute of Bioscience and Biotechnology.

### Dye leakage assay

Calcein-entrapped LUVs composed of EYPE/EYPG (7:3, w/w) were prepared by vortexing the dried lipid in dye buffer solution (70 mM calcein, 10 mM Tris, 150 mM NaCl, 0.1 mM EDTA, pH 7.4). The suspension was subjected to 10 freeze-thaw cycles in liquid nitrogen and extruded 21 times through polycarbonate filters (two stacked 100-nm pore size filters) with a LiposoFast extruder (Avestin, Inc., Canada). Untrapped calcein was removed by gel filtration on a Sephadex G-50 column. The concentration of calcein-entrapped LUVs was determined in triplicate by phosphorus analysis. Calcein leakage from LUVs was monitored at 20 °C by measuring fluorescence intensity at an excitation wavelength of 490 nm and emission wavelength of 520 nm on a model RF-5301PC spectrophotometers. Complete dye release was obtained using 0.1% Triton X-100.

### Membrane depolarization assay

The cytoplasmic membrane depolarization activity of the *r*-RTSN was measured using the membrane potential sensitive dye, DiSC3(5), as previously described^59^,^60^. Briefly, *Staphylococcus aureus* (KCTC 1621) purchased from KCTC was grown at 37 °C with agitation until the mid-log phase (OD_600_ = 0.4) and was harvested by centrifugation. Cells were washed twice with washing buffer (20 mM glucose, 5 mM HEPES, pH 7.4) and resuspended to an OD_600_ of 0.05 in the washing buffer. The cell suspension was incubated with 20 nM DiSC3(5) until stable reduction of fluorescence was achieved, implying incorporation of the dye into the bacterial membrane. Then, KCl was added to a final concentration of 100 mM to equilibrate K^+^ levels. Membrane depolarization was monitored by recording changes in the intensity of fluorescence emission of the membrane potential-sensitive dye, DiSC3(5) (excitation wavelength = 622 nm, emission wavelength = 670 nm) after peptide addition. The membrane potential was fully dissipated by adding gramicidin D (final concentration of 0.2 nM).

## Additional Information

**How to cite this article:** Min, H. J. *et al*. Rattusin structure reveals a novel defensin scaffold formed by intermolecular disulfide exchanges. *Sci. Rep.* **7**, 45282; doi: 10.1038/srep45282 (2017).

**Publisher's note:** Springer Nature remains neutral with regard to jurisdictional claims in published maps and institutional affiliations.

## Supplementary Material

Supplementary Materials

## Figures and Tables

**Table 1:** NMR restraints and structural statistics s for *r-*RTSN.

NMR restraints	No. of restraints
Nuclear Overhauser effect (NOE)-derived distance restraints	722
Hydrogen bond restraints	20
Dihedral angle restraints	40
**Structural statistics (20 structures)**	
Violations	
Number of distance restraints >0.5 Å	0
Number of dihedral angle restraints >5°	0
Root-mean-square deviation (RMSD) from experiments	
Distance (Å)	0.100 ± 0.005
Dihedral angle (°)	2.223 ± 0.340
RMSD from idealized geometry	
Bonds (Å)	0.005 ± 0.000
Angles (°)	0.654 ± 0.026
Impropers (°)	0.497 ± 0.027
Ramachandran analysis (%) (all residues)	
Most favored region	62.1
Allowed region	32.7
Disallowed region	5.2
Average pairwise RMSD (Å) (residues 8–22)	
Backbone heavy atoms	0.39 ± 0.16
All heavy atoms	1.27 ± 0.24
