# Supplementary material for: Rattusin structure reveals a novel defensin scaffold formed by intermolecular disulfide exchanges
Source: Sci Rep. 2017 Mar 27;7:45282. doi: 10.1038/srep45282 (PMC5366907; doi:10.1038/srep45282)
Supplement: Supplementary Materials [file srep45282-s1.pdf]

Rattusin structure reveals a novel defensin scaffold formed by intermolecular disulfide exchanges

Hye Jung Min[1,2], Hyosuk Yun[1], Sehyeon Ji[1], Ganesan Rajasekaran[3], Jae Il Kim[4], Jeong-Sun Kim[1], Song Yub Shin[3*], and Chul Won Lee[1*]

Residue	Atom	Chemical shift
**2 Arg**	2.H	8.70
	2.HA	4.42
	2.HB2	1.76
	2.HB3	1.76
	2.HG2	1.56
	2.HG3	1.63
	2.HD2	3.13
	2.HD3	3.13
**3 Val**	3.H	8.30
	3.HA	4.09
	3.HB	1.99
	3.HG21	0.90
	3.HG22	0.90
	3.HG23	0.90
	3.HG11	0.90
	3.HG12	0.90
	3.HG13	0.90
**4 Arg**	4.H	8.50
	4.HA	4.33
	4.HB2	1.72
	4.HB3	1.78
	4.HG2	1.55
	4.HG3	1.62
	4.HD2	3.14
	4.HD3	3.14
**5 Arg**	5.H	8.45
	5.HA	4.42
	5.HB2	1.74
	5.HB3	1.74
	5.HG2	1.56
	5.HG3	1.56
**6 Thr**	6.H	8.29
	6.HA	4.30
	6.HB	4.12
	6.HG21	1.17
	6.HG22	1.17
	6.HG23	1.17
**7 Leu**	7.H	8.35
	7.HA	4.40
	7.HB2	1.57
	7.HB3	1.57
	7.HG	1.57
	7.HD11	0.88
	7.HD12	0.88
	7.HD13	0.88
	7.HD21	0.83
	7.HD22	0.83
	7.HD23	0.83
**8 Gln**	8.H	8.45
	8.HA	4.36
	8.HB2	1.93
	8.HB3	2.04
	8.HG2	2.31
	8.HG3	2.31
**9 Cys**	9.H	8.55
	9.HA	4.78
	9.HB2	3.03
	9.HB3	3.08
**10 Ser**	10.H	8.73
	10.HA	4.58
	10.HB2	3.81
	10.HB3	3.81
**11 Cys**	11.H	8.44
	11.HA	4.79
	11.HB2	2.96
	11.HB3	3.19
**12 Arg**	12.H	8.53
	12.HA	4.31
	12.HB2	1.76
	12.HB3	1.84
	12.HG2	1.60
	12.HG3	1.67
	12.HD2	3.10
	12.HD3	3.10
**13 Arg**	13.H	8.45
	13.HA	4.29
	13.HB2	1.74
	13.HB3	1.79
	13.HG2	1.56
	13.HG3	1.62
	13.HD2	3.15
	13.HD3	3.15
**14 Val**	14.H	7.96
	14.HA	4.18
	14.HB	2.12
	14.HG21	0.91
	14.HG22	0.91
	14.HG23	0.91
	14.HG11	0.91
	14.HG12	0.91
	14.HG13	0.91
**15 Ser**	15.H	8.16
	15.HA	4.43
	15.HB2	3.90
	15.HB3	3.90
**16 Arg**	16.H	8.43
	16.HA	4.18
	16.HB2	1.85
	16.HB3	1.85
	16.HG2	1.62
	16.HG3	1.622
**17 Asn**	17.H	8.34
	17.HA	4.80
	17.HB2	2.87
	17.HB3	2.77
**18 Thr**	18.H	7.92
	18.HA	4.36
	18.HB	4.22
	18.HG21	1.16
	18.HG22	1.16
	18.HG23	1.16
**19 Cys**	19.H	8.34
	19.HA	4.72
	19.HB2	3.12
	19.HB3	3.12
**20 Ser**	20.H	8.80
	20.HA	4.53
	20.HB2	3.84
	20.HB3	3.84
**21 Cys**	21.H	8.41
	21.HA	4.75
	21.HB2	3.06
	21.HB3	3.06
**22 Ile**	22.H	8.10
	22.HA	4.13
	22.HB	1.83
	22.HD11	0.87
	22.HD12	0.87
	22.HD13	0.87
	22.HG21	0.87
	22.HG22	0.87
	22.HG23	0.87
**23 Arg**	23.H	8.30
	23.HA	4.33
	23.HB2	1.73
	23.HB3	1.81
	23.HG2	1.55
	23.HG3	1.61
	23.HD2	3.13
	23.HD3	3.13
**24 Leu**	24.H	8.24
	24.HA	4.37
	24.HB2	1.63
	24.HB3	1.63
	24.HG	1.59
	24.HD11	0.84
	24.HD12	0.84
	24.HD13	0.84
	24.HD21	0.88
	24.HD22	0.88
	24.HD23	0.879
**25 Ser**	25.H	8.26
	25.HA	4.43
	25.HB2	3.86
	25.HB3	3.86
**26 Arg**	26.H	8.33
	26.HA	4.35
	26.HB2	1.76
	26.HB3	1.88
	26.HG2	1.61
	26.HG3	1.61
	26.HD2	3.16
	26.HD3	3.16
**27 Ser**	27.H	8.30
	27.HA	4.47
	27.HB2	3.79
	27.HB3	3.84
**28 Thr**	28.H	8.04
	28.HA	4.27
	28.HB	4.12
	28.HG21	1.09
	28.HG22	1.09
	28.HG23	1.09
**29 Tyr**	29.H	8.09
	29.HA	4.59
	29.HB2	2.89
	29.HB3	3.07
**30 Ala**	30.H	8.16
	30.HA	4.35
	30.HB1	1.35
	30.HB2	1.35
	30.HB3	1.35
**31 Ser**	31.H	7.83
	31.HA	4.22
	31.HB2	3.83
	31.HB3	3.83

Table S1. Proton chemical shifts (ppm) of the refolded C15S rattusin mutant.

Table S2. Summary of CYANA calculation of *r*-RTSN containing three different disulfide bond connectivities.

	Name	***Assigned peaks*** *(total 842 NOE peaks)*	***Long-range distance constraints***	***Cyana target function***	***Backbone RMSD*** *(only structural region, residues 8-22)*
**(I)**	F4-1 (Intra SS)	741	88	0.13	3.81
**(II)**	F4-2 (Inter SS, Fig. S7a)	750	84	1.71	0.11
**(III)**	F4-2 (Inter SS, Fig. S7b)*	762	93	0.67	0.30

* The preferred disulfide bond structure of *r*-RTSN determined in this study.

a

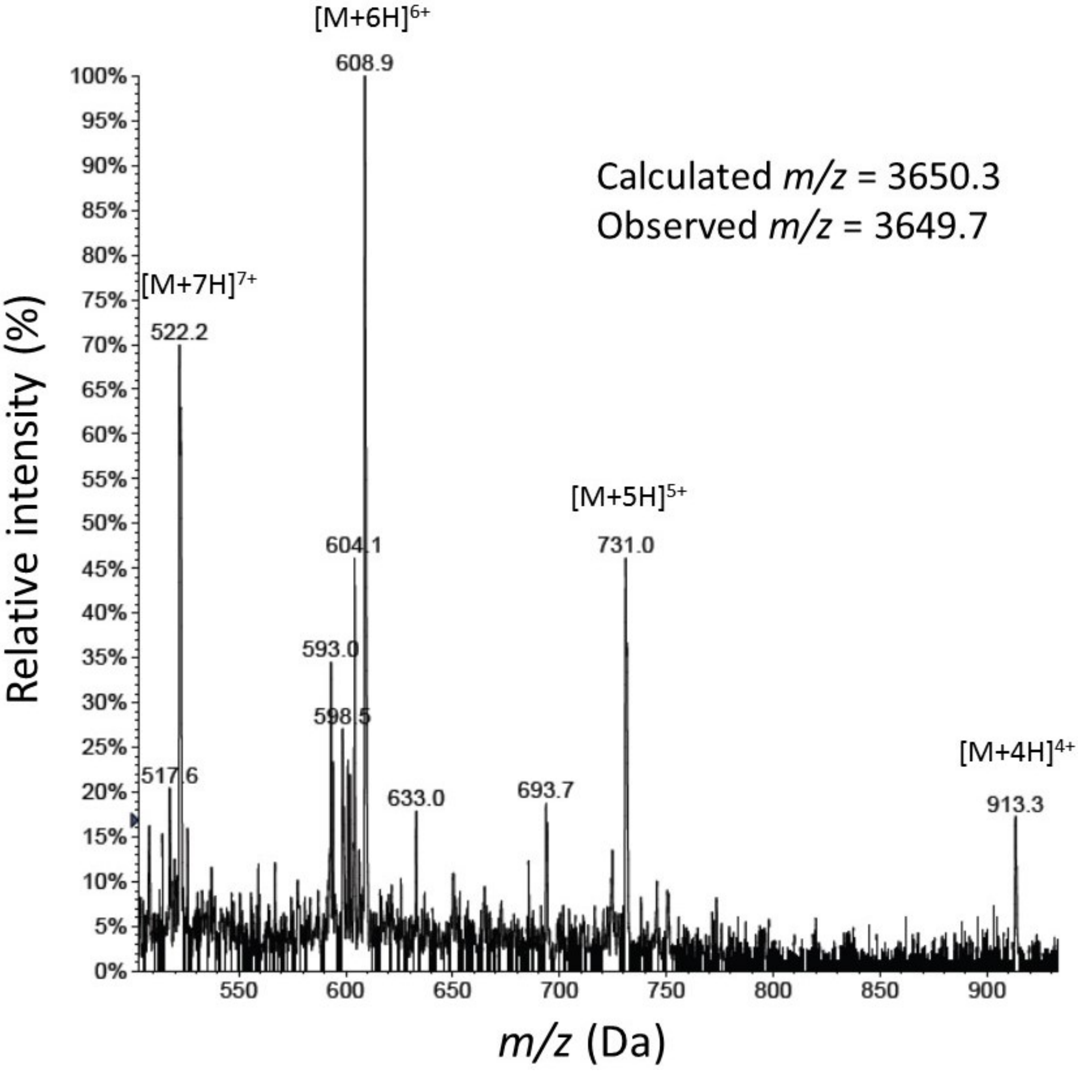

b

		[M+H]$^{1+}$	[M+2H]$^{2+}$	[M+3H]$^{3+}$	[M+4H]$^{4+}$	[M+5H]$^{5+}$	[M+6H]$^{6+}$	[M+7H]$^{7+}$	[M+8H]$^{8+}$	[M+9H]$^{9+}$
m/z	**Linear**	3650.3	1825.7	1217.4	913.3	730.9	609.2	522.3	457.2	406.5
	***r*-RTSN**	7289.6	3645.3	2430.5	1823.2	1458.7	1215.8	1042.2	912.1	810.8

Fig. S1. ESI-LC-MS ionization pattern of linear rattusin (a) and lists of ions and calculated masses of linear and refolded RTSN (b).

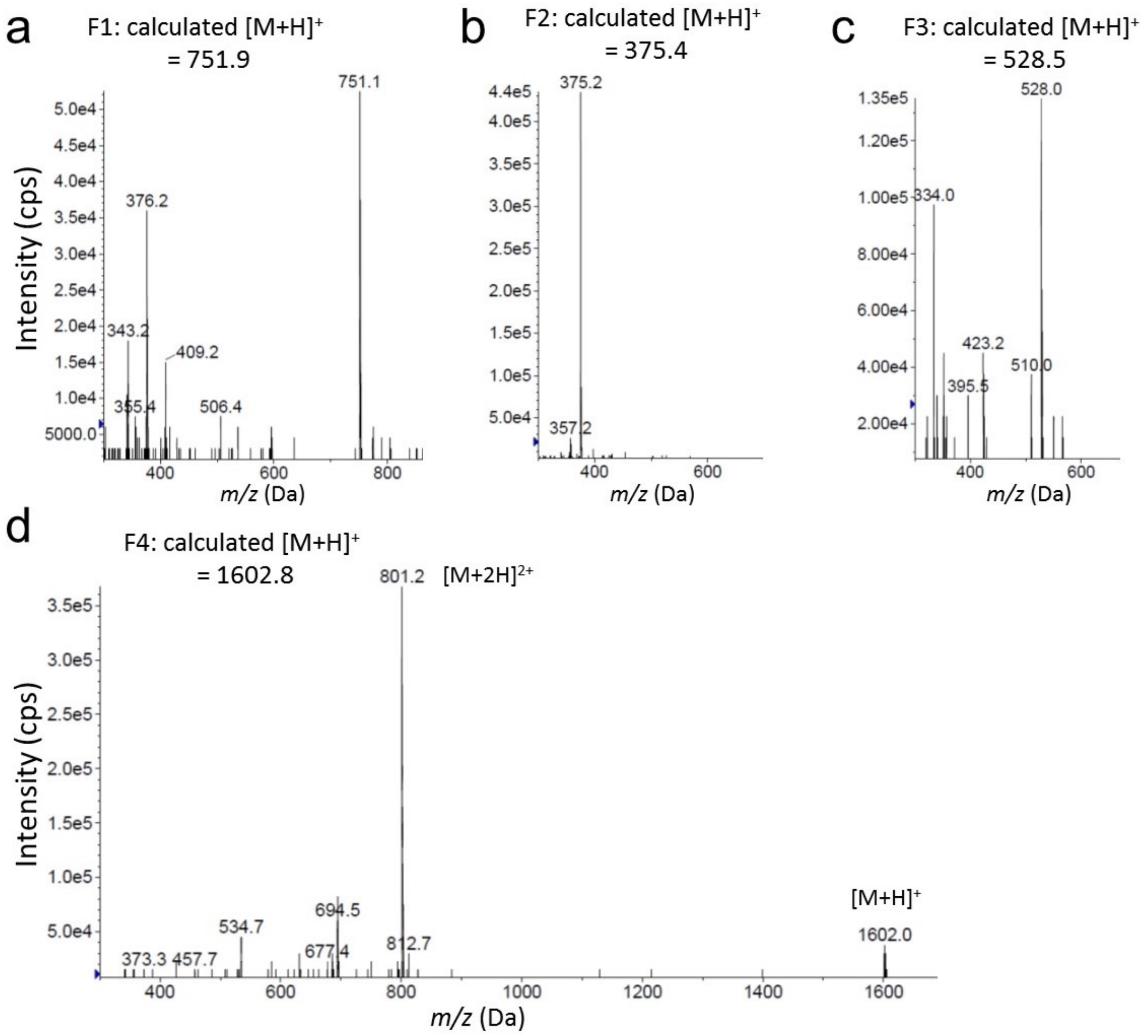

Fig. S2. ESI-LC-MS ionization pattern of trypsin-digested fragments of refolded rattusin (*r*-RTSN). (a) F1 fragment, (b) F2 fragment, (c) F3 fragment, and (d) F4 fragment. Calculated masses (Da) are indicated.

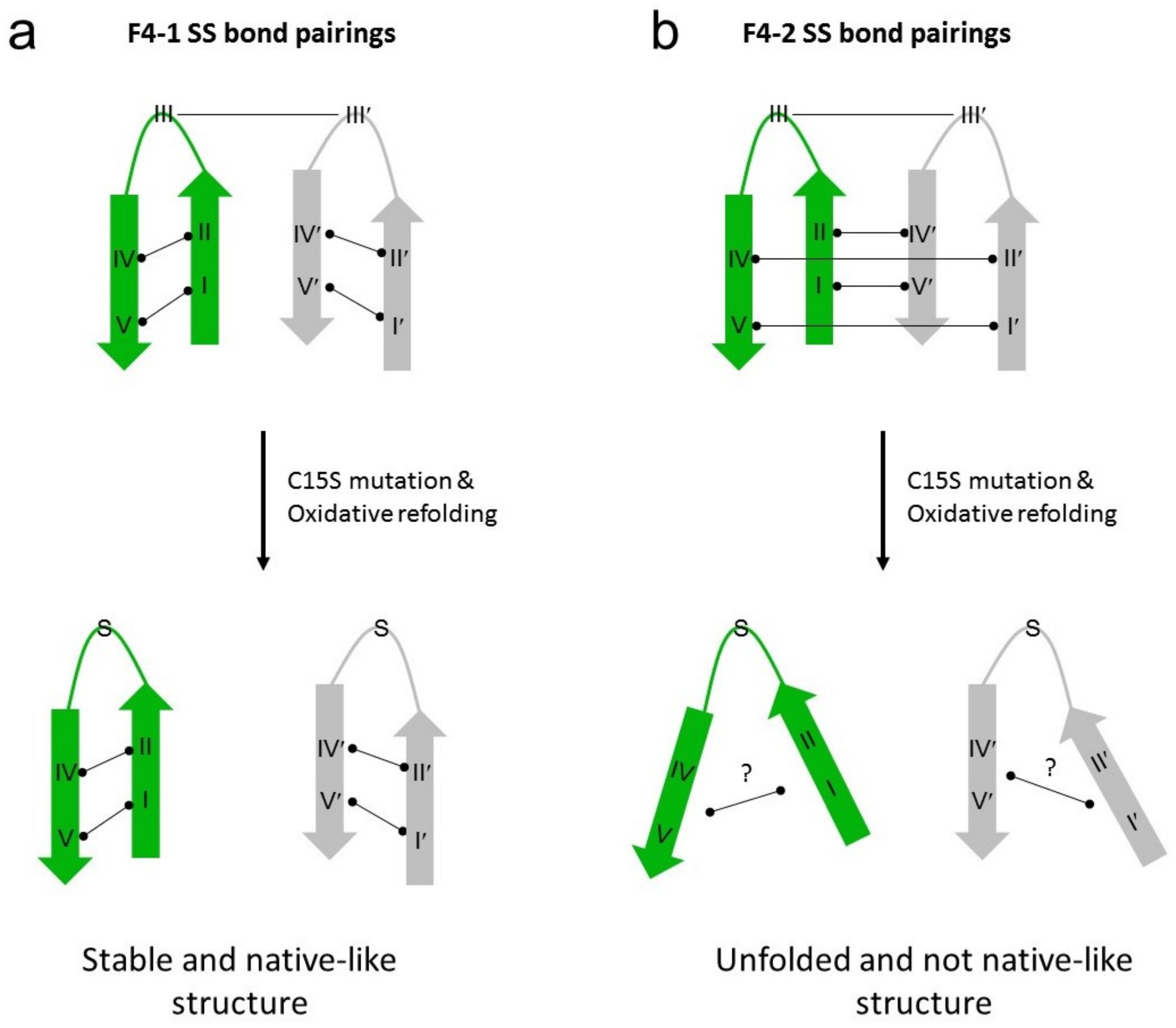

Fig. S3. Schematic diagrams of the SS bond pairings of the F4 fragment and the proposed structure of the C15S mutant. (a) F4-1 SS bond pairings in the refolded rattusin (*r*-RTSN) structure and the proposed monomeric structure of the refolded C15S mutant. (b) F4-2 SS bond pairings in *r*-RTSN structure and the proposed monomeric structure of refolded C15S mutant.

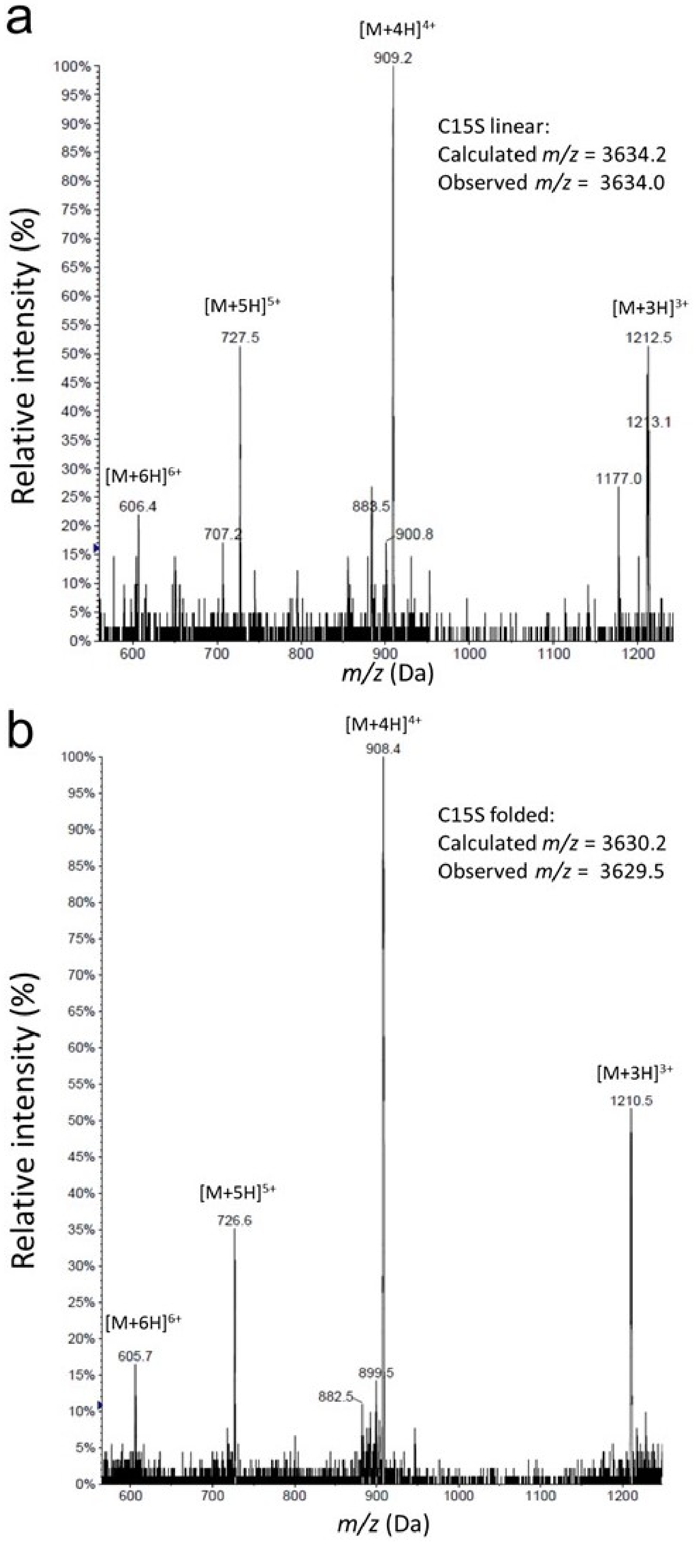

Fig. S4. ESI-LC-MS ionization pattern of linear rattusin (a) and the refolded (b) C15S mutant.

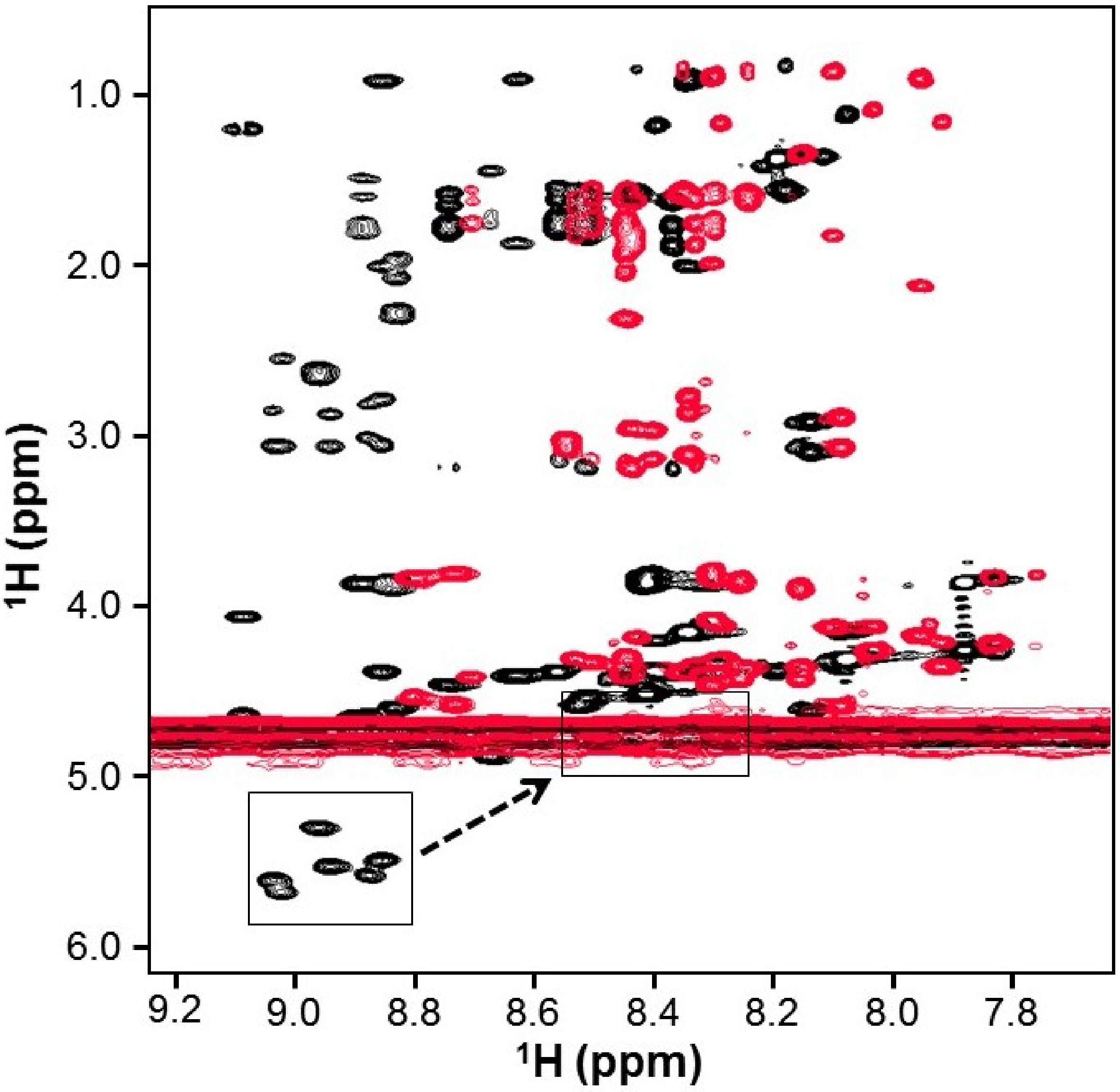

Fig. S5. TOCSY spectra of refolded rattusin (*r*-RTSN) (black) and the refolded C15S mutant (red). The squares indicate the cross peaks of NH-C$^{\alpha}$H of cysteines, which were shifted to the random coil chemical shift region in the refolded C15S mutant spectrum. The dotted arrow indicates the peak shifts.

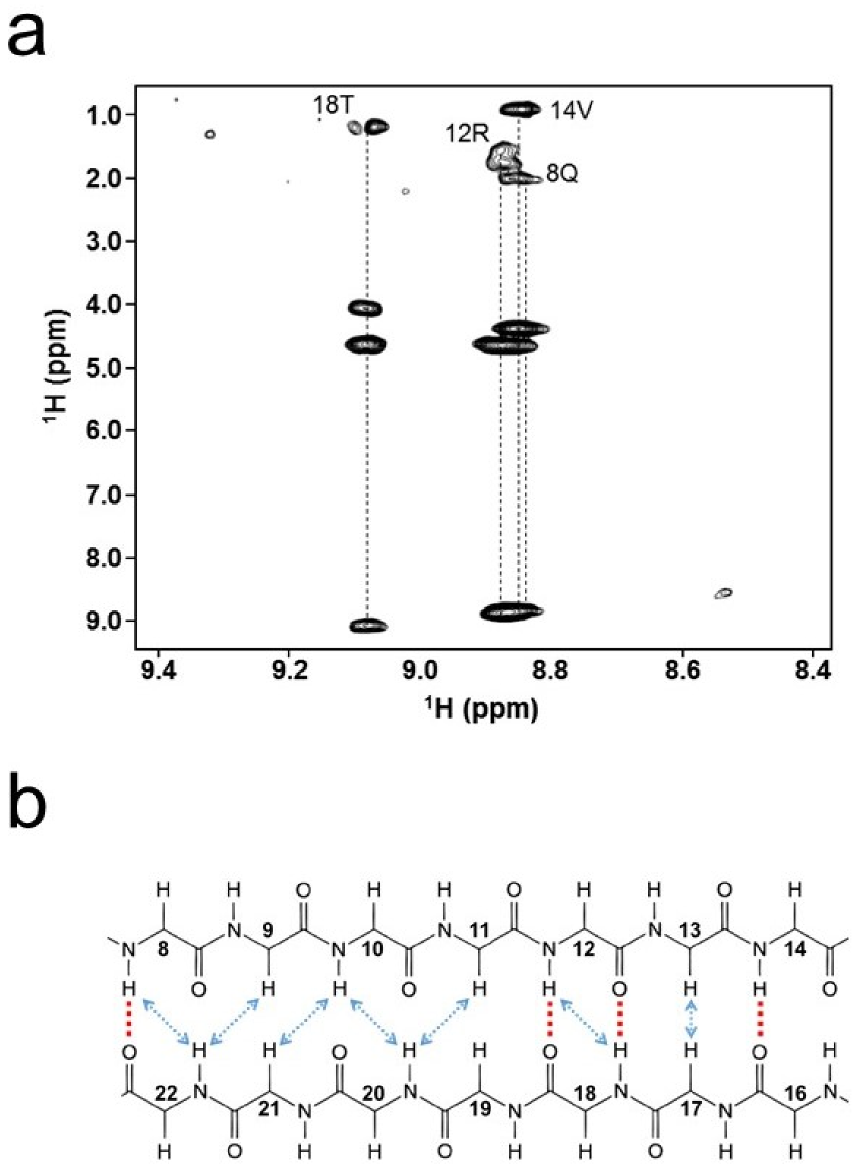

Fig. S6. Hydrogen/deuterium (H/D) exchange experiment (a) and β-hairpin region of the *r*-RTSN (b). (a) TOCSY spectrum of *r*-RTSN was recorded in 40 min after deuterium adding at 25 °C. The non-exchanged peaks were indicated. (b) Hydrogen bonds based on H/D exchange experiment and observed NOEs are indicated as red dotted and blue arrow lines, respectively.

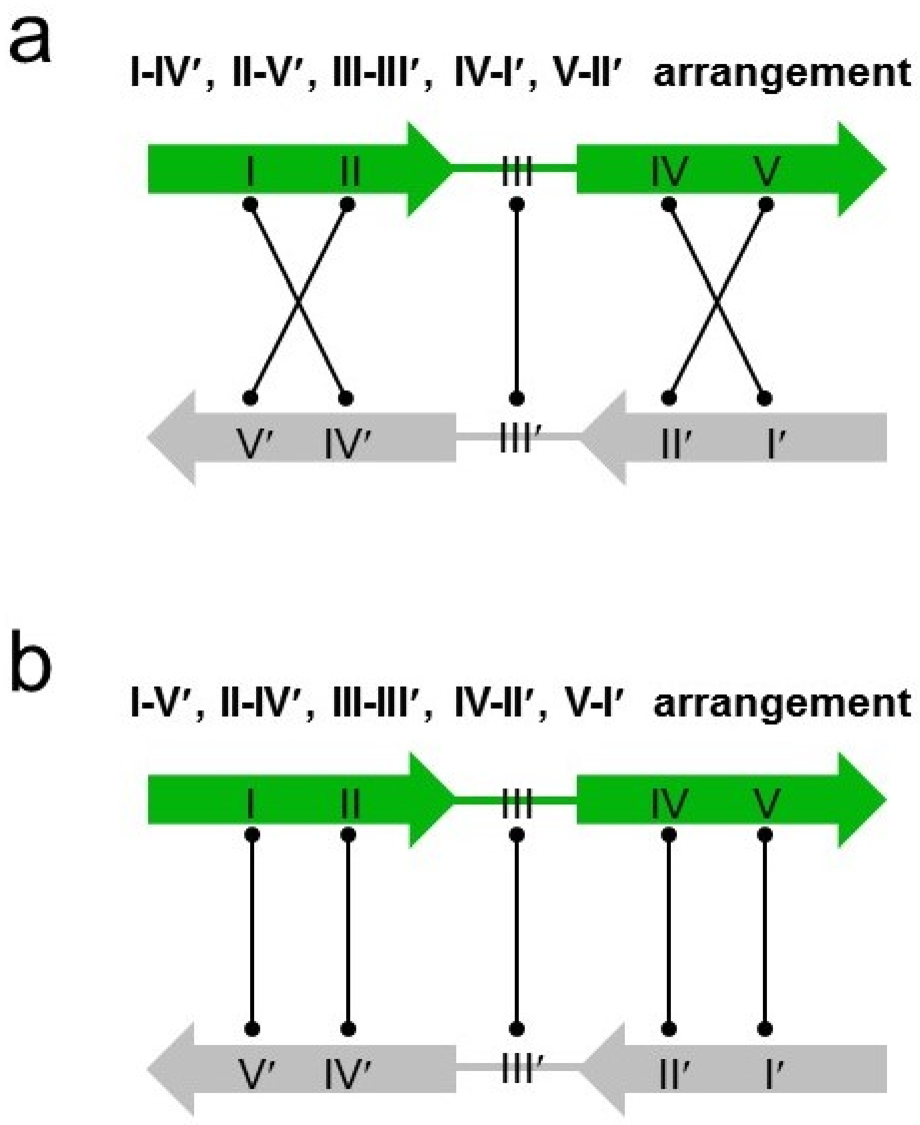

Fig. S7. Schematic diagrams of proposed intermolecular SS bond pairings in F4-2 fragment. (a) The SS bonds are in an I-IV', II-V', III-III', IV-I', V-II' arrangement. (b) The SS bonds are in an I-V', II-IV', III-III', IV-II', V-I' arrangement.

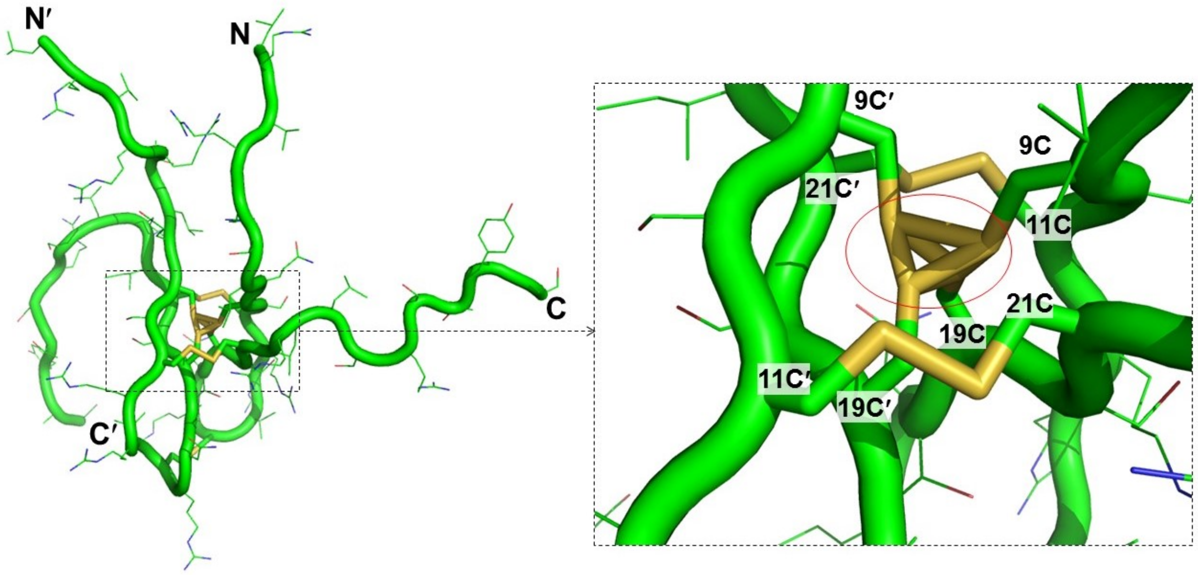

Fig. S8. Cyana model structure of rattusin containing the disulfide bonds (I-IV′, II-V′, III-III′, IV-I′, and V-II′). Core structure formed by disulfide bonds shown as yellow sticks. Red circle indicates the clash of disulfide bonds formed by 9C, 19C, 9C′, and 19C′.

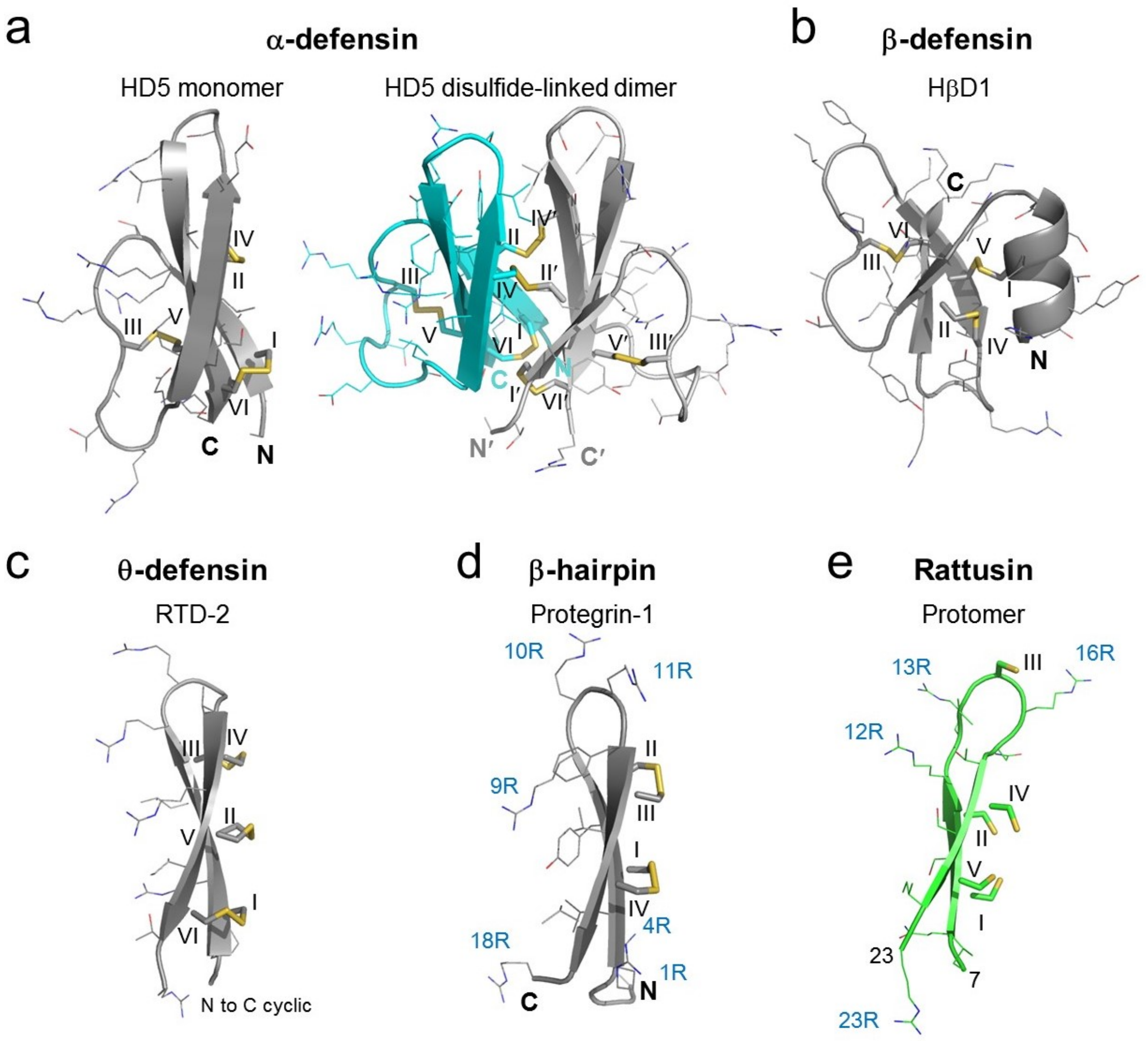

Fig. S9. Ribbon structures of α-defensin HD5 (a), β-defensin HβD1 (b), and θ-defensin RTD-2 (c), β-hairpin protegrin-1 (d), and protomer of refolded rattusin (*r*-RTSN) (e). Cysteine residues are labeled with Roman numerals. Positively charged residues in protegrin-1 (d) and the protomer of *r*-RTSN (e) are labeled.
